# Current Role of Brivaracetam in the Management of Epilepsy in Adults and Children: A Systematic Review

**DOI:** 10.7759/cureus.73413

**Published:** 2024-11-10

**Authors:** Nirmal Surya, Ish Anand, Kanharam N Patel, Abhishek Tandayam, Snehal S Muchhala, Bhavesh P Kotak

**Affiliations:** 1 Neurology, Surya Neuro Centre, Indian Federation of Neurorehabilitation (IFNR), Mumbai, IND; 2 Neurology, Ganga Ram Institute of Postgraduate Medical Education &amp; Research, New Delhi, IND; 3 Neurology, Dr. Reddy’s Laboratories, Hyderabad, IND; 4 Pharmacology, Dr. Reddy's Laboratories, Hyderabad, IND; 5 Medical Affairs, Dr. Reddy’s Laboratories, Hyderabad, IND

**Keywords:** brivaracetam, epilepsy, safety, seizures, treatment outcome

## Abstract

Epilepsy, a neurological condition, has a devastating effect on the quality of life (QoL) of patients if left untreated. Brivaracetam (BRV), a third-generation antiepileptic drug (AED), acts by modulating synaptic vesicle proteins, making it a valuable addition to the arsenal of drugs for epilepsy management. This study aims to assess the efficacy, safety, and reasons for switching from prior AEDs to BRV in patients with epilepsy. A systematic electronic search was performed in PubMed and Google Scholar for English-language articles published from 1 June 2013 to 2 June 2023 on the safety, efficacy, and behavioral adverse effects (BAEs) of BRV when used as monotherapy, add-on therapy, and after switching from prior AEDs (switch therapy; along with reasons for switching to BRV from prior AEDs in adult and pediatric populations), irrespective of the route of administration. A qualitative assessment was conducted using the Joanna Briggs Institute (JBI) tool. A qualitative synthesis of the data was performed. Sixty-one articles involving a total of 15,186 patients with epilepsy were included for qualitative synthesis. In adults, seizure reduction was reported in 31.4%-72.0%, 4.4%-82.1%, and 6.8%-54.3% of patients; seizure freedom in 12.10%-25.6%, 2.0%-80%, and 6.5%-30.6% of patients; and a responder rate of ≥50% in 30.8%, 21.9%-83.8%, and 16.7%-69.1% of patients with monotherapy, add-on therapy, and after switch therapy, respectively. In the pediatric population, seizure reduction was reported in 39.1%‍-‍62.5% and 21%-59% of patients, seizure freedom in 4.4%-37.5% and 12% of patients, and a responder rate of ≥50% in 19.7%-65% and 21%-45.2% of patients with add-on therapy and after switch therapy, respectively. BAEs such as irritability, mood changes, emotional lability, aggression, and agitation were reported in adults for all types of therapies, while anger was reported with only monotherapy and add-on therapy, hyperactivity with add-on therapy, and agitation with monotherapy and add-on therapy with BRV. In the pediatric population, irritability and aggression were reported with add-on and switch therapies, while emotional lability was reported with only switch therapy with BRV. The reasons for switching to BRV from previous AEDs were lack of efficacy and treatment-related adverse effects (AEs). BRV has a favorable efficacy and safety profile. The drug reduces seizure frequency, provides seizure freedom, and achieves a ≥50% responder rate in adult and pediatric patients with add-on therapy and after switching to BRV from other AEDs. However, there is limited evidence supporting its use as monotherapy.

## Introduction and background

Epilepsy is a chronic multifactorial neurological condition characterized by hyperexcitability of neurons, causing recurrent seizures and leading to cognitive and behavioral changes [1,2]. Globally, the condition ranks as the fifth most common neurological disorder after stroke, migraine, dementia, and meningitis [3], with disease-specific disability-adjusted life years of 13 million per year [4]. In India, epilepsy is the third most common debilitating disorder contributing to disability after stroke and headaches [5]. Epilepsy can be focal, generalized, a combination of generalized and focal, and/or of unknown type with immune, genetic, infectious, structural, metabolic, and/or unknown etiological factors [6]. The global prevalence of lifetime epilepsy is estimated to be 7.60 per 1000 persons, with an annual cumulative incidence of 67.77 per 100,000 persons [7]. In India, the estimated prevalence of epilepsy as per 2019 data was 10.1 million [5]. Focal seizures are the most common type in both children and adults [8]. Although the condition affects people of all age groups, it exhibits a bimodal distribution based on age, with peaks observed in younger individuals and in the elderly [9].

The landscape of epilepsy treatment has evolved significantly, encompassing pharmaceutical agents, implantable devices, and surgical interventions. Antiepileptic drugs (AEDs) remain the cornerstone of epilepsy treatment, and about 70% of patients achieve effective seizure control through medication alone [10]. Most of these drugs target the γ-aminobutyric acid (GABA) system, calcium channels, or sodium channels, yet more than 30% of patients fail to achieve prolonged seizure freedom of having no seizures for at least one year or three times the largest pretreatment gap between seizures [11-13].

Treatment recommendations according to the National Institute for Health and Care Excellence (NICE) 2022 guidelines for the different types of seizures are presented in Table 1 [14].

**Table 1:** Treatment recommendations according to NICE 2022 guidelines. LEV: levetiracetam; BRV: brivaracetam; NICE: National Institute for Health and Care Excellence.

Seizure type	First-line monotherapy	Second-line monotherapy	Third-line monotherapy	First-line add-on therapy	Second-line add-on therapy	Third-line add-on therapy
Generalized tonic-clonic seizures	Sodium valproate (boys and men, girls <10 years of age, unlikely to need treatment during childbearing age, women unable to conceive) lamotrigine, LEV (women with the ability to conceive)	Lamotrigine and LEV (if treatment with sodium valproate is unsuccessful)	-	Clobazam, lamotrigine, LEV, perampanel, topiramate, sodium valproate (except in women with the ability to conceive)	BRV, lacosamide, phenobarbital, primidone, zonisamide	-
Focal seizures	Lamotrigine, LEV	Carbamazepine, oxcarbazepine, zonisamide	Lacosamide	Carbamazepine, lacosamide, lamotrigine, LEV, oxcarbazepine, topiramate, zonisamide	BRV, cenobamate, eslicarbazepine acetate, perampanel, pregabalin, sodium valproate (except in women with the ability to conceive)	Phenobarbital, phenytoin, tiagabine, vigabatrin
Absence seizures	Ethosuximide	Sodium valproate (boys and men, girls <10 years of age, unlikely to need treatment during childbearing age, women unable to conceive)	Lamotrigine, LEV	Sodium valproate as add-on therapy (boys and men, girls <10 years of age, unlikely to need treatment during childbearing age, women unable to conceive) Consider lamotrigine or LEV (if treatment with sodium valproate is unsuccessful)	-	-
Myoclonic seizures	Sodium valproate (boys and men, girls <10 years of age, unlikely to need treatment during childbearing age, women unable to conceive). LEV (women with the ability to conceive)	LEV (if treatment with sodium valproate is unsuccessful), consider BRV, clobazam, clonazepam, lamotrigine, phenobarbital, piracetam, topiramate, zonisamide (if treatment with LEV is unsuccessful)	-	LEV (as add-on therapy) Consider BRV, clobazam, clonazepam, lamotrigine, phenobarbital, piracetam, topiramate, zonisamide (if treatment with LEV is unsuccessful)	-	-
Tonic or atonic seizures	Sodium valproate (boys and men, girls <10 years of age, unlikely to need treatment during childbearing age, women unable to conceive). Lamotrigine (women with the ability to conceive)	Lamotrigine (if treatment with sodium valproate is unsuccessful), consider clobazam, rufinamide, or topiramate (if treatment with lamotrigine is unsuccessful)	-	Lamotrigine (add-on therapy), consider clobazam, rufinamide, or topiramate (if treatment with lamotrigine is unsuccessful)	-	-

Despite the effectiveness of AEDs, they are frequently associated with drug-related AEs. The common AEs of these treatments include sedation, dizziness, nausea, ataxia, loss of appetite, fatigue, paradoxical hyperactivity, hypersensitivity, impaired cognitive function and language fluency, hepatotoxicity, leukopenia, gingival hyperplasia, and behavioral problems [10]. Of these, the behavioral adverse effects (BAEs) associated with AEDs are often overlooked; however, these should be given significant consideration. Aggression, hyperactivity, psychosis, agitation, and restlessness are the common BAEs associated with AEDs [15]. Recently introduced AEDs include brivaracetam (BRV), cenobamate, everolimus, cannabidiol, and fenfluramine [16]. BRV is a Food and Drug Administration (FDA)-approved drug, and according to the 2023 prescribing information, BRV is indicated as both monotherapy and adjunct therapy in patients aged one month and older [17,18]. BRV may have a lower likelihood of inducing irritability and other BAEs than levetiracetam (LEV) [19,20]. Nevertheless, the available evidence in this regard remains inconclusive, primarily due to the absence of randomized head-to-head trials directly comparing these two AEDs [20]. Additionally, the use of BRV as monotherapy is not yet approved in all countries. In India, the use of BRV is approved as an adjunctive therapy for focal seizures in patients aged 16 years and older [21]. Considering this, evidence-based global data on the effectiveness and safety of BRV as monotherapy and adjunctive therapy in adults and pediatric patients would help clinicians position BRV in managing epilepsy in the Indian context. This systematic review was conducted to assess the effectiveness and safety of BRV when used as monotherapy, add-on therapy, or after switching from prior AEDs, especially LEV, in adults and pediatric patients.

## Review

Methodology

The review was conducted in accordance with the Preferred Reporting Items for Systematic Reviews and Meta-Analyses (PRISMA) statement and is registered in the International Prospective Register of Systematic Reviews (PROSPERO) with registration number CRD42023425067.

Literature Search

**Figure 1:**
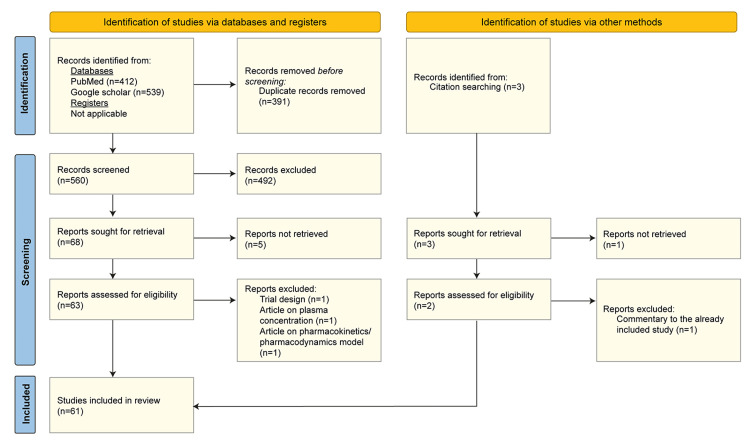
PRISMA flowchart representing the screening process of articles. PRISMA: Preferred Reporting Items for Systematic Reviews and Meta-Analyses.

A comprehensive literature search was performed in the PubMed-MEDLINE and Google Scholar (first 200 articles). Cross-references of the relevant articles and grey literature were also searched. For the PubMed-MEDLINE search, relevant medical subject headings (MeSH) terms and free text terms related to safety, efficacy, epilepsy, and BRV were used along with Boolean operators to frame search strategies according to the review objectives (Supplementary Table 4). The search was conducted by one author, the gathered results were deduplicated, and further screening was performed independently by two authors based on the title and abstract. Any disagreement between the authors at each step was resolved by discussion with a third reviewer. In the next step, the full text of the screened articles was obtained, and a final decision was made on its inclusion/exclusion based on the eligibility criteria. The screening process is presented in a PRISMA flow diagram (Figure 1) [22].

Eligibility Criteria

Comparative clinical trials, single-arm intervention studies, real-world studies, post-market studies, observational studies, and descriptive cross-sectional studies assessing the safety, efficacy, and BAEs of BRV when used as an early or late add-on, as a monotherapy, and after switching to BRV from previous AEDs, especially LEV with reasons reported for switching, were included in the review. There were no restrictions with respect to the route of administration of BRV and the age of patients. Articles published in English over the past 10 years (from 1 June 2013 to 2 June 2023) were considered for review. The exclusion criteria comprised data reported in case studies, review articles, in vitro studies, animal studies, or editorials.

Data Collection and Data Items

Data were extracted and tabulated in a standardized data extraction form prepared in Microsoft Excel. Initially, two-three pilot entries were made independently by two authors, and the disagreement was resolved by discussing with the third author. The data from the studies were extracted based on (1) metadata (first author name, title, publication year, database, and article link); (2) study details (study design and primary and secondary objectives); (3) patient characteristics (epilepsy type, age, and male/female); (4) treatment details (AEDs used, monotherapy/adjunct therapy, treatment duration, and dosage/route of administration); (5) efficacy details (seizure frequency, seizure freedom, responder rate, quality of life (QoL), and other findings); (6) reason for the switch to BRV (in case of studies with switch data); (7) BAEs (irritability, mood changes, hyperactivity, emotional liability, anger, aggression, agitation, and other findings); (8) patients who discontinued using BRV; (9) reason for the discontinuation of BRV; (10) additional remarks (if any); and (11) study conclusion.

Quality Assessment

The eligible studies were assessed for their quality based on the Joanna Briggs Institute (JBI) tool for analytical studies, the JBI tool for randomized controlled trials (RCTs) [23,24], and the JBI tool for quasi-experimental studies. Each study was scored individually. Scores were then consolidated by scoring either 1 (for “yes” and “not applicable” options) or 0 (for “unclear,” “cannot determine,” and “not reported” options). The sum of the points awarded to each question was divided by the highest possible score (eight in the case of analytical studies, 13 in the case of RCTs, and nine in the case of quasi-experimental studies). Studies were considered to be of low quality if the scores were 0-0.3, moderate quality if the scores were 0.4-0.6, and high quality if the scores were 0.7-1.0.

Results

Search Results

The initial search in the database and Google Scholar yielded a total of 951 articles. Additionally, three articles were added through citation tracking. Of the 954 articles, 61 were retrieved for full-text analysis and included in the qualitative synthesis, as shown in the PRISMA flowchart (Figure 1) [22]. All the included studies were either of high quality or moderate quality. Eight studies were of high quality, and one study was of moderate quality per the JBI tool for RCTs, 43 studies were of high quality per the JBI tool for analytical cross-sectional studies, and nine studies were of high quality per the JBI tool for quasi-experimental studies (Supplementary Tables 5-7) [11,12,25-83].

Study and Patient Characteristics

The characteristics of the included studies are summarized in Table 2 [11,12,25-83], and the characteristics of the patients included in the studies are summarized in Table 3.

**Table 2:** Characteristics of the included studies. IV: intravenous; LTFU: long-term follow-up; N: number of patients; NR: not reported; OLE: open-label extension; PBO: placebo. NA (Not applicable): Do not qualify for any specific clinical trial phase due to their study design and focus on observing the patients over time.

First author and year	Study design	Study phase	Epilepsy type	Patient population	Treatment duration
Van Paesschen, 2013 [33]	Phase 2b, double-blind, randomized, placebo‑controlled, parallel-group, dose‑ranging study	Phase 2	Partial	Adult, N=157	2.3 months
Kwan, 2014 [25]	Phase 3, prospective, multicenter, randomized, double-blind, placebo-controlled, parallel-group, flexible-dose trial	Phase 3	Focal epilepsy, generalized epilepsy	Adult, N=480	3.7 months
Ryvlin, 2014 [28]	Phase 3, double-blind, randomized, placebo-controlled, fixed-dose trial	Phase 3	Complex focal seizures, secondarily generalized focal seizures, simple focal seizures, generalized seizures, unclassifiable seizures, seizure clusters	Adult, N=298	2.8 months
Biton, 2014 [27]	Phase 3, prospective, multicenter, randomized, double-blind, placebo-controlled, parallel‑group, fixed-dose, confirmatory trial	Phase 3	Partial onset seizures, complex partial seizures, simple partial seizures, secondarily generalized seizures, generalized seizures, and unclassifiable seizures	Adult, N=298	2.8 months
Klein, 2015 [30]	Randomized, double-blind, placebo-controlled, multicenter study	Phase 3	Focal epilepsy, epileptic syndrome	Adult, N=760	2.8 months
Yates, 2015 [80]	Phase 3b, open-label, single-arm, prospective, multicenter study	Phase 3	Partial onset seizures, simple partial seizures, complex partial seizures, partial evolving to secondarily generalized seizures, primary generalized seizures, absence, atypical absence, myoclonic, clonic, tonic-clonic, atonic, unclassifiable	Adult, N=29	2.8 months
Klein, 2016 [31]	Phase 3, multicenter, randomized, four-arm, parallel-group study	Phase 3	Focal seizures, simple, complex, secondary generalized, generalized seizures, absence, tonic-clonic, and NR	Adult and pediatric, N=105	1.3 months
Steinig, 2017 [75]	Multicenter, retrospective cohort study	NA	Focal, idiopathic generalized, symptomatic generalized, and unclassified epilepsy	Adult, N=253 Pediatric, N=9	12 months
Steinhoff, 2017 [38]	Monocenter survey	NA	Partial onset and generalized tonic-clonic seizures	Adult, N=101	6 months
Strzelczyk A, 2017 [77]	Cohort study	NA	Generalized tonic-clonic status epilepticus, nonconvulsive status epilepticus, simple-partial, and complex-partial	Adult, N=11	NR
Zahnert, 2018 [63]	Retrospective study	NA	Idiopathic generalized	Adult, N=93	NR
Arnold, 2018 [32]	Two double-blind, randomized, multicenter, historical control, phase 3 studies	Phase 3	NR	Adult, N=150	3.9 months
Willems, 2018 [74]	Multicenter, retrospective cohort study	NA	Lennox-Gastaut syndrome, tuberous sclerosis complex, Unverricht-Lundborg syndrome, and continuous spike waves in sleep	Adult, N=35 Pediatric, N=9	6.9 months
Kalss, 2018 [70]	Retrospective, single-center study	NA	Tonic‐clonic status epilepticus, myoclonic status epilepticus with coma, epilepsia partialis continua, aphasic status, and aura continua	Adult, N=7	NR
Schubert-Bast, 2018 [46]	Multicenter, retrospective study	NA	Focal onset seizures with preserved awareness, focal onset seizures with impaired awareness, focal to bilateral tonic-clonic seizures, and other	Pediatric, N=34	5.9 months
Witt, 2018 [54]	Retrospective study	NA	Simple-partial, complex-partial, and generalized	Adult, N=43	0.16–5.7 months
Hirsch, 2018 [36]	Monocenter, retrospective, outcome analysis	NA	Focal and/or structural, genetic progressive myoclonus, and unknown	Adult, N=102	9.9 months
Strzelczyk, 2018 [37]	Multicenter, retrospective cohort study	NA	Juvenile myoclonic epilepsy, childhood absence epilepsy, Jeavons syndrome, myoclonic-astatic epilepsy, and genetic generalized epilepsy	Adult, N=49 Pediatric, N=12	7–24 months
Villanueva, 2019 [42]	Multicenter retrospective study	NA	Simple partial, complex partial seizure, secondary generalized	Adult, N=575	12 months
Theochari, 2019 [64]	Retrospective study	NA	Focal, generalized, unclassified	Adult, N=25	8.5 months
Menzler, 2019 [35]	Retrospective, observational, multicenter study	NA	Genetic generalized epilepsy	Adult, N=615	26.3 months
Liu, 2019 [78]	Phase 2a, open-label, single-arm, fixed three-step, dose-escalation trial	Phase 2	Focal, generalized, unclassified	Pediatric, N=99	0.7 months
Nissenkorn, 2019 [43]	Cross-sectional, retrospective chart review	NA	Focal onset and epileptic syndrome	Pediatric, N=31	6.7 months
Santamarina, 2019 [55]	Retrospective, observational multicenter study	NA	Tonic‐clonic status epilepticus, myoclonic status epilepticus, focal motor status epilepticus, epilepsia partialis continua, adversive status epilepticus, oculoclonic status epilepticus, ictal paresis, tonic status epilepticus, generalized (absence status epilepticus), focal without impairment of consciousness, aphasic status epilepticus, and focal with impaired consciousness	Adult, N=44	NR
Adewusi, 2020 [52]	Multicenter retrospective study	NA	Focal, generalized, unclassified	Adult, N=290	12 months
Lerche, 2020 [61]	Multicenter, retrospective, noninterventional chart review	NA	All seizures	Adult, N=506	6 months
Lafortune, 2020 [40]	Retrospective, two-center study	NA	Focal, primary generalized	Adult, N=38	8.25 months
Visa-Rene, 2020 [45]	Observational and retrospective study	NA	Epileptic encephalopathy, focal epilepsy, epilepsy with generalized tonic-clonic seizures, and childhood absence epilepsy	Pediatric, N=46	12 months
Liguori, 2020 [69]	Retrospective, double-center study	NA	Focal genetic, focal structural, focal unknown, generalized genetic, combined generalized and focal	Adult, N=43	12 months
Klein, 2020 [26]	Randomized, double-blind, PBO-controlled phase 3 trial	Phase 3	Focal seizure	Adult, N=503	2.8 months
Steinhoff, 2020 [68]	Prospective, noninterventional, post-marketing study	NA	All seizures	Adult, N=266	6 months
Fonseca, 2020 [73]	Retrospective cohort study	NA	Juvenile myoclonic epilepsy, generalized tonic-clonic seizures alone, juvenile absence, myoclonic absence epilepsy, childhood absence epilepsy, and Jeavons syndrome	Adult, N=37	10.4 months
Arnold, 2020 [82]	Open-label, multicenter, single-arm, LTFU trial	Phase 3	Focal seizure: Partial seizures, simple partial seizures, complex partial seizures, partial evolving to secondary generalized, and generalized seizures	Adult, N=108	3–90 months
O’Brien, 2020 [81]	Phase 3, multicenter, open-label, flexible-dose, uncontrolled, long-term follow-up trial	Phase 3	Focal seizures	Adult, N=616 Pediatric, N=51	48–132 months
Szaflarski, 2020 [29]	Phase 2, open-label, randomized, active‑control, proof-of-concept trial	Phase 2	Partial seizures and generalized seizures	Adult, N=30	At least 1 dose
McGuire, 2020 [39]	Retrospective chart review	NA	Focal epilepsy, generalized epilepsy, and mixed	Pediatric, N=23	8.2 months
Stephen, 2021 [12]	Prospective audit	NA	Focal seizure, juvenile myoclonic epilepsy, generalized tonic-clonic seizures	Adult, N=108	6 months
Lattanzi, 2021 [50]	Retrospective, multicenter study	NA	Focal onset, focal to bilateral tonic-clonic	Adult, N=1029	12 months
Lattanzi, 2021 [71]	Real-world time-based analysis	NA	Focal onset, focal to bilateral tonic-clonic	Adult, N=387	10.3 months
Depondt, 2021 [67]	Retrospective, longitudinal, multicenter chart review	NA	Uncontrolled focal seizures	Adult, N=175	9 months
Russo, 2021 [41]	Retrospective study	NA	Epileptic encephalopathy and refractory focal epilepsies	Pediatric, N=8	8 months
Savastano, 2021 [62]	Observational study	NA	Focal epilepsy	Adult, N=76	6 months
Ferretjans, 2021 [34]	Retrospective and descriptive study	NA	Generalized epilepsy, focal, and multifocal	Pediatric, N=66	16.6 months
Orlandi, 2021 [48]	Retrospective, observational, multicenter study	NA	Acute symptomatic, remote symptomatic, progressive symptomatic, cryptogenic, multifactorial, and post-anoxic status epilepticus	Adult, N=56	NR
Strzelczyk, 2021 [72]	Retrospective, multicenter study	NA	Focal epilepsy, genetic generalized epilepsy, and unclassified epilepsy syndromes	Adult, N=253 Pediatric, N=9	19.2 months
Toledo, 2021 [79]	Open-label, multicenter, flexible-dose, single-arm trial	Phase 3	Focal (partial onset), focal aware (simple partial), focal impaired awareness (complex partial), focal to bilateral tonic-clonic (partial evolving to secondary generalized), generalized onset seizures, absence, tonic, and tonic-clonic	Adult, N=749	At least 36 months
Abraira, 2021 [60]	Retrospective descriptive study	NA	Focal structural epilepsy, generalized genetic epilepsy, unclassified epilepsy, and unknown epilepsy	Adult, N=41	12 months
Ben-Menachem, 2021 [11]	Phase 3, multicenter, single-arm, long-term follow-up, OLE trial	Phase 3	Focal seizures, generalized onset seizures, and Unverricht-Lundborg disease	Adult, N=839 Pediatric, N=14	168 months
Stefanatou, 2021 [66]	Retrospective, observational, multicenter study	NA	Focal seizures, generalized seizures, and unclassified seizures	Adult, N=156	NR
Lattanzi, 2022 [47]	Retrospective study	NA	Focal onset, focal to bilateral tonic-clonic	Adult, N=1029	12 months
Russo, 2022 [51]	Multicenter, retrospective, observational study	NA	Focal generalized, epileptic encephalopathy	Pediatric, N=45	At least 1 month
Lattanzi, 2022 [44]	Retrospective study	NA	Focal onset, focal to bilateral tonic-clonic	Adult, N=1029	12 months
Lattanzi, 2022 [58]	Retrospective, multicenter study	NA	Focal onset, bilateral tonic-clonic	Adult, N=176	12 months
Snoeren, 2022 [65]	Retrospective study	NA	Focal, generalized, combined generalized and focal, unknown	Adult, N=379	20 months
Svendsen, 2022 [56]	Retrospective study	NA	Focal, generalized, and unclassified	Adult, N=120	>12 months
Farkas, 2022 [76]	Phase 2, multicenter, open-label trial	Phase 2	NR	Pediatric, N=50	1.6 months
Green, 2022 [53]	Observational study	NA	Focal aware, focal impaired awareness, focal to bilateral, generalized motor, tonic-clonic, tonic-atonic, myoclonic, generalized nonmotor (absence), and other	Adult, N=200	0.06–74.3 months
Lattanzi, 2022 [59]	Retrospective study	NA	Focal onset, focal to bilateral tonic-clonic	Adult, N=994	12 months
Orlandi, 2023 [49]	Retrospective, observational, multicenter study	NA	Focal, generalized, unknown	Adult, N=97	0.09 months
Naddell, 2023 [57]	Open-label, single-center, observational study	NA	Focal epilepsy, generalized epilepsy, combined focal, generalized onset	Adult, N=109	12.6 months
Chavarría, 2023 [83]	Prospective, unblinded, monocentric study	Phase 2	Focal onset, focal aware, focal impaired awareness, and focal to bilateral tonic-clonic	Adult, N=11	1 dose of 10-minute IV infusion

**Table 3:** Characteristics of patients in the included studies. AED: antiepileptic drug; BRV: brivaracetam; LEV: levetiracetam. ^a^Epilepsia partialis continua, adversive status epilepticus, oculoclonic status epilepticus, ictal paresis, epileptic encephalopathy, and/or combination of epilepsy or with unknown/unclassified epilepsy. ^b^Carbamazepine, clobazam, eslicarbazepine, ethosuximide, lacosamide, lamotrigine, oxcarbazepine, perampanel, topiramate, valproate, and zonisamide.

Characteristic	Overall population (N=15,186)
Age, range	11.4 months–62.3 years
Sex, n (%)	
Male	7447 (49.0%)
Female	7739 (51.0%)
Epilepsy type, n (%)	
Focal epilepsy	8714 (57.3)
Genetic generalized epilepsy	688 (4.5)
Tonic-clonic status epilepticus	66 (0.4)
Myoclonic status epilepticus	56 (0.36)
Focal motor status epilepticus	5 (0.03)
Others^a^	5657 (37.2)
BRV line of treatment, n (%)	
First‑line	1620 (10.7)
Second‑line	4531 (29.8)
Not reported	9035 (59.4)
Switch from prior AEDs, n (%)	3232 (21.3)
Switch from LEV	1972 (61.0)
Switch from other prior AEDs^b^	1260 (39.0)

Eight studies employed an open-label design, 16 studies used a placebo-controlled design, seven studies reported using a blinded design, and 30 studies utilized a retrospective cross-sectional design. The majority of the included studies were published in 2019-2021 (n=19). About 15,186 patients with epilepsy received BRV as monotherapy or add-on therapy or switched to BRV from previous AEDs. The sample sizes ranged from 7 to 1029 across the included studies. Some studies were conducted selectively on pediatric patients (n=9), the majority on adult patients (n=45), and some on both populations (n=7). The mean age across the studies ranged from 11.4 months to 62.3 years. The study duration ranged from 0.09 months to 168 months. The male-to-female ratio of patients with epilepsy ranged from 0.17 to 2.10. Focal epilepsy was the most common type of epilepsy reported among the patients. Other types of epilepsy reported in the studies were genetic generalized epilepsy, tonic-clonic status epilepticus, myoclonic status epilepticus, focal motor status epilepticus, epilepsia partialis continua, adversive status epilepticus, oculoclonic status epilepticus, ictal paresis, epileptic encephalopathy, and a combination of epilepsy types and unknown/unclassified epilepsy. The route of BRV administration was reported to be oral in 11 studies (dose: 20.1-200 mg/day) and intravenous in 10 studies (dose: 100‍-‍200 mg); the other studies did not provide data on the route of BRV administration. A majority of the patients (61.0%) switched to BRV from LEV.

Efficacy

Reduction in seizure frequency: Among adult patients, a reduction in seizure frequency after the administration of BRV as monotherapy, add-on therapy, and after switching from prior AEDs was observed in 31.4%-72.0% of patients (n=2 studies), 4.4%-82.1% of patients (n=20 studies), and 6.8%-54.3% of patients (n=11 studies), respectively. After switching to BRV, the seizure frequency was reported to increase in 2.1%‍-‍21.7% of patients (n=8 studies) or remained unchanged in 15%-61.0% of patients (n=5 studies). With add-on therapy, the seizure frequency was reported to increase in 2.2%-16.6% of patients (n=2 studies).

Among pediatric patients, a reduction in seizure frequency after the administration of BRV as an add-on therapy and after switching from prior AEDs was seen in 39.1%-62.5% of patients (n=4 studies) and in 21%-59% of patients (n=2 studies), respectively. After switching to BRV, the seizure frequency was reported to increase in 7.1%-19.3% of patients (n=2 studies). With add-on treatment, the seizure frequency remained unchanged in 40.9%-55% of patients (n=2 studies) and was reported to increase in 4.3% of patients (n=1 study).

Seizure freedom: Among adult patients, seizure freedom was achieved in 12.10%-25.6% of patients (n=2 studies) who received BRV monotherapy, in 2.0%-80% of patients (n=25 studies) who received BRV as add-on therapy, and in 6.5%-30.6% of patients (n=13 studies) who switched to BRV from prior AEDs. Seizure freedom was maintained for 6-12 months in patients who received monotherapy, 2.7-12 months in patients who received add-on therapy, and 6-12 months in patients who switched to BRV from prior AEDs.

Among pediatric patients, seizure freedom was achieved in 4.4%-37.5% of patients (n=5 studies) who received BRV as add-on therapy and in 12% of patients (n=1 study) who switched to BRV from prior AEDs. Seizure freedom was maintained up to 12 months in patients who switched to BRV from prior AEDs. The seizure-free duration was not reported in monotherapy and add-on therapy groups in studies on pediatric patients.

Responder rate: Among adult patients, a responder rate of ≥50% (the proportion of patients with a 50% or greater decline in seizure frequency during a defined period of treatment) was achieved in 30.8% of patients (n=1 study) who received BRV monotherapy, in 21.9%-83.8% of patients (n=20 studies) who received BRV as add-on therapy, and in 16.7%-69.1% of patients (n=12 studies) who switched to BRV from prior AEDs.

**Figure 2:**
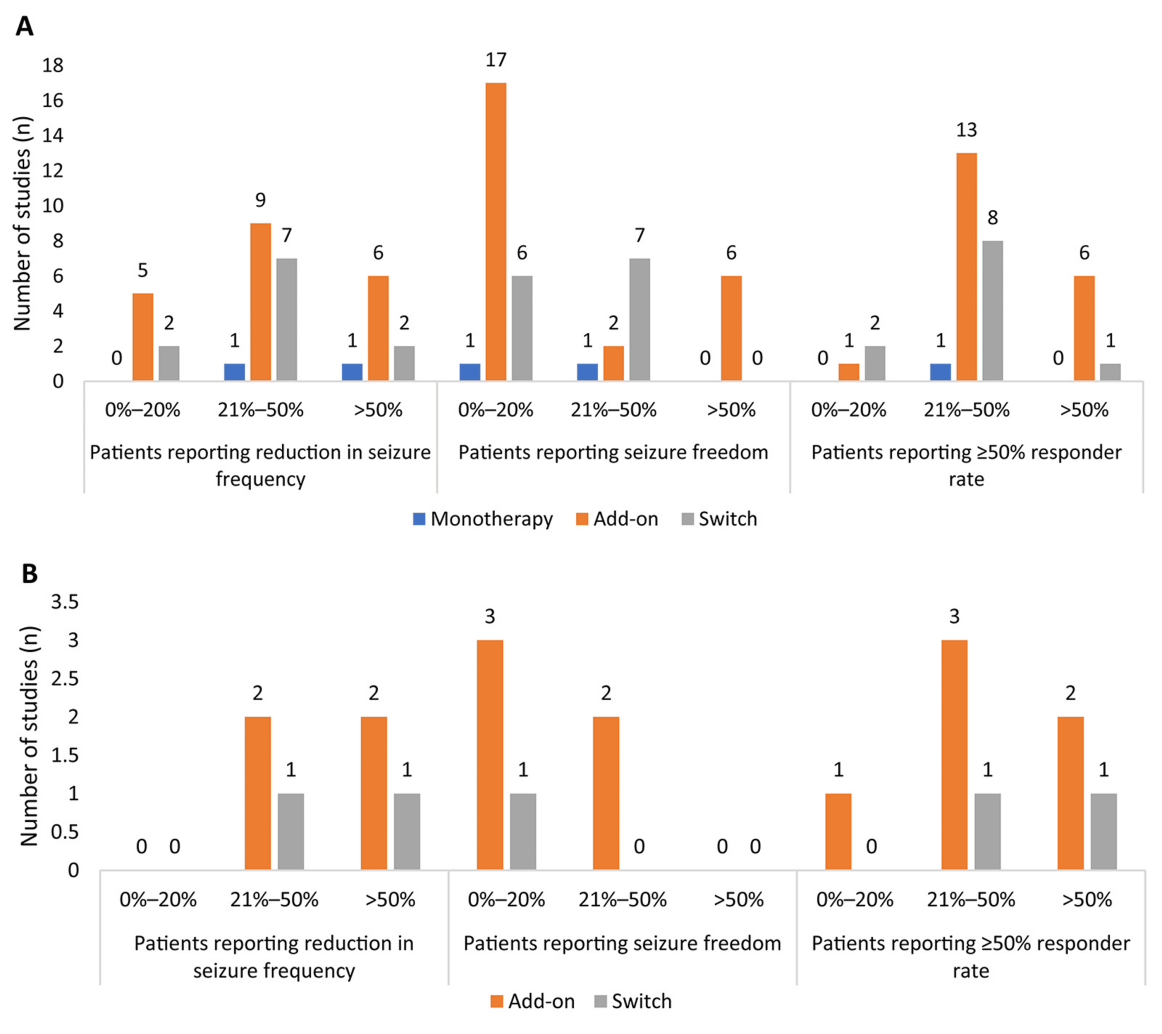
Number of studies in which 0%–20%, 21%–50%, and >50% of the (A) adult and (B) pediatric patients reported a reduction in seizure frequency, seizure freedom, and ≥50% responder rate.

Among pediatric patients, a responder rate of ≥50% was achieved in 19.7%-65% of patients (n=6 studies) who received BRV as add-on therapy and in 21%-45.2% of patients (n=2 studies) who switched to BRV from prior AEDs. Figure 2A, B represents the reduction in seizure frequency, seizure freedom, and responder rate with respect to the number of studies. 

Safety

Behavioral adverse effects: Among adult patients, BAEs reported with BRV administration were identified as irritability, mood changes, hyperactivity, emotional lability, aggression, and agitation. With BRV monotherapy, irritability was reported in 1.3%-29.4% of patients (n=3 studies), mood changes were reported in 0.6%-28.4% of patients (n=3 studies), emotional lability in 10.1%-16.7% of patients (n=2 studies), anger in 0.9%‍-‍18.3% of patients (n=2 studies), aggression in 0.9%-11.9% of patients (n=2 studies), and agitation in 2.8% of patients (n=1 study). Hyperactivity was not reported among patients who received BRV monotherapy. When administered as add-on therapy, irritability was reported in 0.2%-52.0% of patients (n=11 studies), mood changes in 0.7%-9.1% of patients (n=11 studies), hyperactivity in 31.6% of patients (n=1 study), emotional lability in 0.8%-28.9% of patients (n=9 studies), anger in 0.8%‍-‍23.7% of patients (n=4 studies), aggression in 1%-25% of patients (n=13 studies), and agitation in 3.1%-5.9% of patients (n=4 studies). Among patients who switched to BRV from prior AEDs, irritability was reported in 2.8%-10% of patients (n=7 studies), mood changes were reported in 0.9%‍-‍23% of patients (n=5 studies), emotional lability was reported in 0.9%-6% of patients (n=6 studies), and aggression was reported in 3%‍-‍8.5% of patients (n=3 studies). BAEs, such as anger, hyperactivity, and agitation, were not reported among patients who switched to BRV from previous AEDs.

Among pediatric patients, the most commonly reported BAEs were irritability, emotional lability, and aggression. When administered as add-on therapy, irritability was reported in 12.1%‍-‍17.3% of patients (n=2 studies) and aggression in 2%-2.2% of patients (n=2 studies). BAEs such as mood changes, hyperactivity, emotional lability, and anger were not reported among patients who received BRV as add-on therapy. Among patients who switched to BRV from prior AEDs, emotional lability was reported in 3.2% (n=1 study), and irritability and aggression were reported in 5.9% (n=1 study). BAEs such as mood changes, hyperactivity, and agitation were not reported among patients who switched to BRV from previous AEDs.

Other adverse effects: The most common other AEs reported with BRV administration, irrespective of whether the patients were administered BRV as monotherapy or add-on therapy or if they switched to BRV from prior AEDs, were fatigue, nausea, vomiting, and insomnia. Other AEs include headache, ataxia, weight gain, tremors, decreased appetite, vertigo, constipation, upper respiratory tract infection, nasopharyngitis, influenza, arthralgia, memory disturbance, pyrexia, pruritis, back pain, urinary tract infection, contusion, upper abdominal pain, asthenia, weight loss, and diplopia.

In adults, treatment with BRV was discontinued in 14.8%-27.5% of patients (n=3 studies) after monotherapy, 1.3%-47% of patients (n=27 studies) after add-on therapy, and 1.2%-36% of patients (n=14 studies) after switching to BRV from prior AEDs. In the pediatric population, BRV treatment was discontinued in 6.1%-46.6% of patients (n=4 studies) after add-on therapy and 3.2%-9% of patients (n=2 studies) after switching to BRV from prior AEDs.

Quality of Life

An improvement in QoL was seen in the majority of the studies, irrespective of whether the patients were administered BRV as add-on therapy or if they switched to BRV from prior AEDs. Patient-weighted Quality of Life in Epilepsy Inventory-Form 31 (QOLIE-31-P) was the most common QoL tool used across the studies (n=6 studies). Other tools used were Patient Global Evaluation Scale (P-GES) (n=1 study), Investigator Global Evaluation Scale (I-GES) (n=1 study), Clinical Global Impression of Change (CGIC) (n=1 study), Patient’s Global Impression of Change (PGIC) (n=1 study), EuroQol-5 Dimensions Questionnaire (EQ-5D) (n=1 study), and Quality of Life in Epilepsy-10 (n=1 study). The QOLIE-31-P score increased by 12.1±11.4 from baseline to the follow-up period (n=1 study), reached 6.5 from a baseline score of 5.7 (n=1 study), and demonstrated an improvement among 44.2%-46.8% of patients (n=2 studies). An improvement in QoL measured by the P-GES, I-GES, CGIC, and PGIC scores was also noted in 76.9%, 92.3%, 66.2%, and 56.7% of patients, respectively. The Quality of Life in Epilepsy-10 score improved from 41.4 to 35.1 at follow-up. However, no significant improvement in QoL was reported in one study, while another study demonstrated a decrease in QoL with BRV treatment.

Reasons for Switching to BRV

Lack of efficacy and AEs associated with other prior AEDs were the main reasons for switching to BRV in both adult and pediatric populations. Lack of efficacy in terms of poor seizure control was reported among 12.3%-53.6% of patients (n=6 studies). The AEs associated with other prior AEDs, which were responsible for switching to BRV, were psychiatric and BAEs among 24.4%‍-63.6% of patients (n=13 studies), cognitive disturbance among 4.3% of patients (n=1 study), general physical symptoms among 24.5% of patients (n=1 study), drowsiness among 12.2% of patients (n=1 study), and other reasons such as somnolence, gait ataxia, gastrointestinal problems, and alopecia among 10% of patients (n=1 study).

Discussion

Epilepsy manifests across a range of intensities and consequences, with each individual encountering a unique experience, often accompanied by other coexisting conditions. BRV was found to effectively reduce seizure frequency in adult and pediatric patients, irrespective of whether it was administered as monotherapy or add-on therapy or when patients switched to BRV from prior AEDs. Seizures may occur sporadically for some patients, whereas for others, they can be significantly more frequent, with some individuals experiencing as many as 100 seizures daily [84]. Seizure frequency is reported to be influenced by treatment period, patient age, poor lifestyle and sleep patterns, a history of depression, and the type of treatment provided [85].

According to the NICE 2022 guidelines, sodium valproate, lamotrigine, or LEV is preferred as a first-line choice for generalized tonic-clonic seizures, ethosuximide for absence seizures, sodium valproate or LEV for myoclonic seizures, and carbamazepine, lamotrigine, LEV, oxcarbazepine, topiramate, or zonisamide for focal seizures [14]. In this analysis, BRV use was reported in the management of several types of epilepsies, with the majority of studies including patients with focal epilepsy followed by generalized epilepsy, tonic-clonic epilepsy, and others. While BRV is officially authorized solely for the treatment of focal seizures [18], many studies have demonstrated its efficacy in diminishing seizure occurrence in various other forms of epilepsy as well. BRV has also demonstrated promising results in reducing seizure frequency and achieving seizure freedom with a favorable safety profile in patients with status epilepticus.

In the present analysis, BRV add-on and switch therapy were mostly associated with a moderate reduction in seizure frequency (21%-50%). However, comparative data on the seizure frequency in patients before and after BRV add-on therapy would have allowed a more accurate evaluation of the effect of BRV add-on on seizure frequency. Further, a reduction in seizure frequency with BRV monotherapy was reported in only two studies, highlighting relatively limited evidence of BRV as monotherapy.

Seizure freedom is the primary treatment goal for patients who experience seizures and for medical professionals and healthcare teams who provide care for these patients. Attaining freedom from seizures has the potential to influence not only seizure frequency but also other outcomes that hold significance for patients, including disability, QoL, risk of death from different causes (such as sudden unexpected death in epilepsy (SUDEP)), utilization of healthcare resources, and cost outcomes [86]. In this review, BRV as add-on therapy was found to be beneficial in achieving seizure freedom. A majority of the included studies reported achieving seizure freedom in up to 20% of patients, which was a noteworthy achievement.

The line of therapy has a major influence on the possibility of achieving seizure freedom in patients. Most patients achieve seizure freedom within the first two lines of therapy [87]. In a longitudinal observational cohort study that observed patients with epilepsy in Scotland over 30 years, the rate of seizure freedom for one year was 63.7%, of whom 89.9% of the patients had achieved seizure freedom with first-line or second-line AED regimens [13]. The authors showed that a third AED regimen could increase the probability of achieving one-year seizure freedom by only 4.1% [13]. In this analysis, almost 30% of the study population received BRV as the second or later-line therapy, and there were no data on how many patients used BRV in the third or later lines of therapy. Only 10% of patients received the first-line BRV. Therefore, it is important to note that the timing of the initiation of treatment with an AED plays a vital role in the probability of achieving seizure freedom. Seizure freedom rates can also vary depending on various factors, such as age, sex, and type of epilepsy [88,89]. However, no particular association could be drawn in this context because of the wide heterogeneity in the baseline characteristics of the study population in this analysis. Given that there is no universal definition for the effectiveness of an AED in achieving seizure freedom, it remains a challenge to make comparative inferences from clinical trial data.

BRV can cause BAEs, such as irritability, anger, and aggression, with reported real-world incidence rates of 5.6%, 3.3%, and 2.5%, respectively [90]. Evidence from clinical trials as well as from the real-world setting was reviewed in this study, but it must be noted that tolerability data from controlled trials do not adequately consider the presence of comorbidities or other risk factors for these BAEs [90]. This could explain the wide variation in the proportion of patients reporting BAEs, such as irritability, mood changes, hyperactivity, and aggression. Psychiatric and behavioral disorders are seen in almost 39% of patients with epilepsy [91]. Some patients with epilepsy may have a higher predisposition to developing BAEs [92]. Despite the effectiveness of BRV, few patients in the studies included in this analysis discontinued treatment due to a lack of efficacy and AEs such as psychiatric and BAEs, and other effects such as gait ataxia, gastrointestinal problems, and alopecia when treated with BRV, highlighting the role of healthcare practitioners in informing patients about the possible AEs of BRV and establishing regular monitoring and management protocols.

Therefore, the analysis of the temporal association between diagnosis with BAEs and the initiation of treatment with the AED is essential to establish a causal relationship with the AED. In this review, AEs led to treatment discontinuation in 1.2%-47% of patients included in the analysis (n=50 studies). Thus, patients on treatment with BRV should be monitored for AEs. Nevertheless, a meta-analysis reported that despite mild-to-moderate AEs, the tolerability profile of the drug was excellent [93].

Patients with epilepsy have poor QoL due to stigmatization of the condition, disease severity, seizure frequencies, and AEs associated with AEDs [94]. Studies have reported poor QoL in patients managed with conventional AEDs. Phenytoin was associated with worse QoL due to associated AEs [95]. Likewise, carbamazepine demonstrated significantly less improvement in QoL compared with newer AEDs, such as LEV [96]. Several tools are used to assess QoL outcomes in patients with epilepsy, such as QOLIE-31-P, P-GES, I-GES, CGIC, PGIC, EQ-5D, and Quality of Life in Epilepsy-10, with QOLIE-31-P being the most common. In this analysis, BRV administration was associated with an improvement in QoL outcomes irrespective of whether it was administered as monotherapy or add-on therapy or if the patients switched to BRV after receiving other AEDs. The patients demonstrated improvement in cognitive function, conducting daily activities, emotional well-being, level of energy, and overall health status [11,68]. The improved QoL in these patients could probably be attributed to the improvement in seizure frequency with the use of BRV, as reported across the studies.

Although the review offers a robust compilation of efficacy and safety data derived from various study designs, including moderate and high-quality studies, for BRV used as add-on therapy and monotherapy and after switching from other AEDs, there are a few limitations to consider. First, there was a wide variation in the types of seizures included in the studies. Second, there were differences in the characteristics of the populations in each study with respect to age, previous seizure frequency, previous history of AEDs, and associated AEs, leading to heterogeneous results. Third, the sample size across the included studies ranged from seven to 1029 patients, again contributing to a wide variation in the observed results. These variations resulted in a wide range of efficacy and safety outcomes; thus, a meta-analysis is recommended to obtain pooled data on seizure frequency, seizure freedom, ≥50% responder rate, and AEs of BRV. Fourth, the available data did not provide adequate information to draw a substantial conclusion regarding the efficacy and safety of BRV when used as monotherapy. Fifth, the definitions of BAEs, such as irritability, aggression, and anger, were not consistent across the studies, leading to a wide range of reported incidence rates.

## Conclusions

The results of this systematic review of studies that reported the use of BRV for the management of epilepsy in adult and pediatric patients point toward a favorable efficacy and safety profile of BRV as an add-on therapy and after a switch to BRV from other AEDs. However, it also highlights the limited evidence supporting its use as monotherapy. Further studies with large sample sizes are recommended, specifically investigating the utilization of BRV as monotherapy and comparing its treatment outcomes to those observed after switching from previous AEDs.

## Appendices

**Table 4:** Search strings. AED: antiepileptic drugs; BRV: brivaracetam; MeSH: Medical Subject Heading.

Research question	Search details in PubMed
What is the efficacy of brivaracetam after switching from other AEDs in adult patients with epilepsy?	("Brivaracetam"[Title/Abstract] OR "BRV"[Title/Abstract]) AND ("antiepileptic drug*"[Title/Abstract] OR "aed"[Title/Abstract] OR "anticonvulsant*"[Title/Abstract]) AND ("Efficacy"[Title/Abstract] OR "treatment efficacy"[Title/Abstract] OR "efficacy treatment"[Title/Abstract] OR "clinical efficacy"[Title/Abstract] OR "efficacy clinical"[Title/Abstract]) AND ("Epilepsy"[Title/Abstract] OR "epileptic"[Title/Abstract]) AND ("adult"[MeSH Terms] OR "adult"[All Fields] OR "adults"[All Fields] OR "adult s"[All Fields] OR "child"[MeSH Terms] OR "child"[All Fields] OR "children"[All Fields] OR "child's"[All Fields] OR "children's"[All Fields] OR "childrens"[All Fields] OR "childs"[All Fields] OR "paediatrics"[All Fields] OR "pediatrics"[MeSH Terms] OR "pediatrics"[All Fields] OR "paediatric"[All Fields] OR "pediatric"[All Fields]) AND ("switch"[All Fields] OR "switched"[All Fields] OR "switches"[All Fields] OR "switching"[All Fields] OR "switchings"[All Fields]) AND (y_10[Filter])
What is the efficacy of brivaracetam after switching from other AEDs in adult patients with epilepsy?	("Brivaracetam"[Title/Abstract] OR "BRV"[Title/Abstract]) AND ("antiepileptic drug*"[Title/Abstract] OR "aed"[Title/Abstract] OR "anticonvulsant*"[Title/Abstract]) AND ("Efficacy"[Title/Abstract] OR "treatment efficacy"[Title/Abstract] OR "efficacy treatment"[Title/Abstract] OR "clinical efficacy"[Title/Abstract] OR "efficacy clinical"[Title/Abstract]) AND ("Epilepsy"[Title/Abstract] OR "epileptic"[Title/Abstract]) AND ("adult"[MeSH Terms] OR "adult"[All Fields] OR "adults"[All Fields] OR "adult s"[All Fields] OR "child"[MeSH Terms] OR "child"[All Fields] OR "children"[All Fields] OR "child's"[All Fields] OR "children's"[All Fields] OR "childrens"[All Fields] OR "childs"[All Fields] OR "paediatrics"[All Fields] OR "pediatrics"[MeSH Terms] OR "pediatrics"[All Fields] OR "paediatric"[All Fields] OR "pediatric"[All Fields]) AND (y_10[Filter])
What are the behavioral adverse effects associated with the use of brivaracetam when switched from other AEDs?	("Brivaracetam"[Title/Abstract] OR "BRV"[Title/Abstract]) AND ("antiepileptic drug*"[Title/Abstract] OR "aed"[Title/Abstract] OR "anticonvulsant*"[Title/Abstract]) AND ("Epilepsy"[Title/Abstract] OR "epileptic"[Title/Abstract]) AND ("adult"[MeSH Terms] OR "adult"[All Fields] OR "adults"[All Fields] OR "adult s"[All Fields] OR "child"[MeSH Terms] OR "child"[All Fields] OR "children"[All Fields] OR "child's"[All Fields] OR "children's"[All Fields] OR "childrens"[All Fields] OR "childs"[All Fields] OR "paediatrics"[All Fields] OR "pediatrics"[MeSH Terms] OR "pediatrics"[All Fields] OR "paediatric"[All Fields] OR "pediatric"[All Fields]) AND ((((("behavioural adverse event*"[Title/Abstract] OR "behavioral side effect*"[Title/Abstract] OR "behavioural adverse event*"[Title/Abstract] OR "behavioural side effect*"[Title/Abstract]) AND "neuropsychiatric adverse event*"[Title/Abstract]) OR "neuropsychiatric side effect*"[Title/Abstract] OR "psychiatric adverse event*"[Title/Abstract]) AND (("psychiatrical"[All Fields] OR "psychiatrically"[All Fields] OR "psychiatrics"[All Fields] OR "psychiatry"[MeSH Terms] OR "psychiatry"[All Fields] OR "psychiatric"[All Fields]) AND "side"[All Fields] AND "effect*OR"[All Fields] AND "Psychobehavioural"[All Fields] AND "adverse event"[Title/Abstract])) OR ("Psychobehavioral"[All Fields] AND "adverse event"[Title/Abstract]) OR ("Psychobehavioural"[All Fields] AND "side effect*"[Title/Abstract]) OR ("Psychobehavioral"[All Fields] AND "side effect*"[Title/Abstract]) OR "adverse event*"[Title/Abstract] OR "irritability"[Title/Abstract] OR "mood change*"[Title/Abstract] OR "hyperactivity"[Title/Abstract] OR "emotional lability"[Title/Abstract] OR "anger"[Title/Abstract] OR "aggression"[Title/Abstract]) AND ("switch"[All Fields] OR "switched"[All Fields] OR "switches"[All Fields] OR "switching"[All Fields] OR "switchings"[All Fields]) AND (y_10[Filter])
What are the behavioral adverse effects associated with the use of brivaracetam when switched from other AEDs?	("Brivaracetam"[Title/Abstract] OR "BRV"[Title/Abstract]) AND ("antiepileptic drug*"[Title/Abstract] OR "aed"[Title/Abstract] OR "anticonvulsant*"[Title/Abstract]) AND ("Epilepsy"[Title/Abstract] OR "epileptic"[Title/Abstract]) AND ("adult"[MeSH Terms] OR "adult"[All Fields] OR "adults"[All Fields] OR "adult s"[All Fields] OR "child"[MeSH Terms] OR "child"[All Fields] OR "children"[All Fields] OR "child's"[All Fields] OR "children's"[All Fields] OR "childrens"[All Fields] OR "childs"[All Fields] OR "paediatrics"[All Fields] OR "pediatrics"[MeSH Terms] OR "pediatrics"[All Fields] OR "paediatric"[All Fields] OR "pediatric"[All Fields]) AND ((((("behavioural adverse event*"[Title/Abstract] OR "behavioral side effect*"[Title/Abstract] OR "behavioural adverse event*"[Title/Abstract] OR "behavioural side effect*"[Title/Abstract]) AND "neuropsychiatric adverse event*"[Title/Abstract]) OR "neuropsychiatric side effect*"[Title/Abstract] OR "psychiatric adverse event*"[Title/Abstract]) AND (("psychiatrical"[All Fields] OR "psychiatrically"[All Fields] OR "psychiatrics"[All Fields] OR "psychiatry"[MeSH Terms] OR "psychiatry"[All Fields] OR "psychiatric"[All Fields]) AND "side"[All Fields] AND "effect*OR"[All Fields] AND "Psychobehavioural"[All Fields] AND "adverse event"[Title/Abstract])) OR ("Psychobehavioral"[All Fields] AND "adverse event"[Title/Abstract]) OR ("Psychobehavioural"[All Fields] AND "side effect*"[Title/Abstract]) OR ("Psychobehavioral"[All Fields] AND "side effect*"[Title/Abstract]) OR "adverse event*"[Title/Abstract] OR "irritability"[Title/Abstract] OR "mood change*"[Title/Abstract] OR "hyperactivity"[Title/Abstract] OR "emotional lability"[Title/Abstract] OR "anger"[Title/Abstract] OR "aggression"[Title/Abstract]) AND (y_10[Filter])
What are the reasons for switching to brivaracetam from other AEDs?	("Brivaracetam"[Title/Abstract] OR "BRV"[Title/Abstract]) AND ("antiepileptic drug*"[Title/Abstract] OR "aed"[Title/Abstract] OR "anticonvulsant*"[Title/Abstract]) AND ("Epilepsy"[Title/Abstract] OR "epileptic"[Title/Abstract]) AND ("adult"[MeSH Terms] OR "adult"[All Fields] OR "adults"[All Fields] OR "adult s"[All Fields] OR "child"[MeSH Terms] OR "child"[All Fields] OR "children"[All Fields] OR "child's"[All Fields] OR "children's"[All Fields] OR "childrens"[All Fields] OR "childs"[All Fields] OR "paediatrics"[All Fields] OR "pediatrics"[MeSH Terms] OR "pediatrics"[All Fields] OR "paediatric"[All Fields] OR "pediatric"[All Fields]) AND ("switch"[All Fields] OR "switched"[All Fields] OR "switches"[All Fields] OR "switching"[All Fields] OR "switchings"[All Fields]) AND ("reason"[All Fields] OR "reasonable"[All Fields] OR "reasonably"[All Fields] OR "reasons"[All Fields]) AND (y_10[Filter])
What is the efficacy and safety of using brivaracetam when used as a monotherapy in patients with epilepsy?	("adult"[MeSH Terms] OR "adult"[All Fields] OR "adults"[All Fields] OR "adult s"[All Fields] OR "child"[MeSH Terms] OR "child"[All Fields] OR "children"[All Fields] OR "child's"[All Fields] OR "children's"[All Fields] OR "childrens"[All Fields] OR "childs"[All Fields] OR "paediatrics"[All Fields] OR "pediatrics"[MeSH Terms] OR "pediatrics"[All Fields] OR "paediatric"[All Fields] OR "pediatric"[All Fields]) AND ("Epilepsy"[Title/Abstract] OR "epileptic"[Title/Abstract]) AND ("Brivaracetam"[Title/Abstract] OR "BRV"[Title/Abstract]) AND ("Efficacy"[Title/Abstract] OR "treatment efficacy"[Title/Abstract] OR "efficacy treatment"[Title/Abstract] OR "clinical efficacy"[Title/Abstract] OR "efficacy clinical"[Title/Abstract]) AND ("safety"[Title/Abstract] OR "safe"[Title/Abstract]) AND (y_10[Filter])
	("adult"[MeSH Terms] OR "adult"[All Fields] OR "adults"[All Fields] OR "adult s"[All Fields] OR "child"[MeSH Terms] OR "child"[All Fields] OR "children"[All Fields] OR "child's"[All Fields] OR "children's"[All Fields] OR "childrens"[All Fields] OR "childs"[All Fields] OR "paediatrics"[All Fields] OR "pediatrics"[MeSH Terms] OR "pediatrics"[All Fields] OR "paediatric"[All Fields] OR "pediatric"[All Fields]) AND ("Epilepsy"[Title/Abstract] OR "epileptic"[Title/Abstract]) AND ("Brivaracetam"[Title/Abstract] OR "BRV"[Title/Abstract]) AND ("Efficacy"[Title/Abstract] OR "treatment efficacy"[Title/Abstract] OR "efficacy treatment"[Title/Abstract] OR "clinical efficacy"[Title/Abstract] OR "efficacy clinical"[Title/Abstract]) AND (("Efficacy"[Title/Abstract] OR "treatment efficacy"[Title/Abstract] OR "efficacy treatment"[Title/Abstract] OR "clinical efficacy"[Title/Abstract] OR "efficacy clinical"[Title/Abstract] OR "safety"[Title/Abstract] OR "safe"[Title/Abstract]) AND (y_10[Filter])
What is the efficacy of brivaracetam when used as an early or late add-on in patients with epilepsy?	("adult"[MeSH Terms] OR "adult"[All Fields] OR "adults"[All Fields] OR "adult s"[All Fields] OR "child"[MeSH Terms] OR "child"[All Fields] OR "children"[All Fields] OR "child's"[All Fields] OR "children's"[All Fields] OR "childrens"[All Fields] OR "childs"[All Fields] OR "paediatrics"[All Fields] OR "pediatrics"[MeSH Terms] OR "pediatrics"[All Fields] OR "paediatric"[All Fields] OR "pediatric"[All Fields]) AND ("Epilepsy"[Title/Abstract] OR "epileptic"[Title/Abstract]) AND ("Brivaracetam"[Title/Abstract] OR "BRV"[Title/Abstract]) AND ("Efficacy"[Title/Abstract] OR "treatment efficacy"[Title/Abstract] OR "efficacy treatment"[Title/Abstract] OR "clinical efficacy"[Title/Abstract] OR "efficacy clinical"[Title/Abstract]) AND (y_10[Filter])
What are the behavioral adverse effects of brivaracetam when used as an early or late add-on in patients with epilepsy?	("adult"[MeSH Terms] OR "adult"[All Fields] OR "adults"[All Fields] OR "adult s"[All Fields] OR "child"[MeSH Terms] OR "child"[All Fields] OR "children"[All Fields] OR "child's"[All Fields] OR "children's"[All Fields] OR "childrens"[All Fields] OR "childs"[All Fields] OR "paediatrics"[All Fields] OR "pediatrics"[MeSH Terms] OR "pediatrics"[All Fields] OR "paediatric"[All Fields] OR "pediatric"[All Fields]) AND ("Epilepsy"[Title/Abstract] OR "epileptic"[Title/Abstract]) AND ("Brivaracetam"[Title/Abstract] OR "BRV"[Title/Abstract]) AND (("adverse effect*"[Title/Abstract] OR "side effect*"[Title/Abstract] OR "drug related side effects and adverse reactions"[Title/Abstract]) AND (y_10[Filter])

**Table 5:** Quality assessment using the JBI tool for RCTs. JBI: Joanna Briggs Institute; RCTs: randomized controlled trials.

Author	Was true randomization used for assignment of participants to treatment groups?	Was allocation to treatment groups concealed?	Were treatment groups similar at the baseline?	Were participants blind to treatment assignment?	Were those delivering the treatment blind to treatment assignment?	Were treatment groups treated identically other than the intervention of interest?	Were outcome assessors blind to treatment assignment?	Were outcomes measured in the same way for treatment groups?	Were outcomes measured in a reliable way	Was follow-up complete and if not, were differences between groups in terms of their follow-up adequately described and analyzed?	Were participants analyzed in the groups to which they were randomized?	Was appropriate statistical analysis used?	Was the trial design appropriate, and any deviations from the standard RCT design (individual randomization, parallel groups) accounted for in the conduct and analysis of the trial?	Total score/13	Quality interpretation
Paesschen, 2013 [33]	Yes	Yes	Yes	Yes	Yes	Yes	Unclear	Yes	Yes	Yes	Yes	Yes	Yes	0.92	High
Kwan, 2014 [25]	Yes	Unclear	Yes	Yes	Yes	Yes	Unclear	Yes	Yes	No	Yes	Yes	Yes	0.76	High
Biton, 2014 [27]	Yes	Yes	Yes	Yes	Yes	Yes	Unclear	Yes	Yes	Yes	Yes	Yes	Yes	0.92	High
Ryvlin, 2014 [28]	Yes	Yes	Yes	Yes	Yes	Yes	Unclear	Yes	Yes	Yes	Yes	Yes	Yes	0.92	High
Klein, 2015 [30]	Yes	Yes	Yes	Yes	Yes	Yes	Unclear	Yes	Yes	Yes	Yes	Yes	Yes	0.92	High
Klein, 2016 [31]	Yes	Yes	Yes	Yes	Yes	Yes	No	Yes	Yes	Yes	Unclear	Yes	Yes	0.84	High
Arnold, 2018 [32]	Yes	Yes	Unclear	Yes	Yes	Yes	Unclear	Yes	Yes	Yes	Yes	Yes	Yes	0.84	High
Klein, 2020 [26]	Yes	Yes	Yes	Yes	Yes	Yes	Unclear	Yes	Yes	Yes	Yes	Yes	Yes	0.92	High
Szaflarski, 2020 [29]	Yes	No	No	No	No	Yes	No	Yes	Yes	Yes	Yes	Yes	Yes	0.61	Moderate

**Table 6:** Quality assessment using the JBI tool for analytical cross-sectional studies. JBI: Joanna Briggs Institute.

Author	Were the criteria for inclusion in the sample clearly defined?	Were the study subjects and the setting described in detail?	Was the exposure measured in a valid and reliable way?	Were objective, standard criteria used for measurement of the condition?	Were confounding factors identified?	Were strategies to deal with confounding factors stated?	Were the outcomes measured in a valid and reliable way?	Was appropriate statistical analysis used?	Total score/8	Quality interpretation
Steinhoff, 2017 [38]	Yes	Yes	Yes	Yes	No	No	Yes	Yes	0.75	High
Steinig, 2017 [75]	Yes	Yes	Yes	Yes	No	No	Yes	Yes	0.75	High
Hirsch, 2018 [36]	Yes	Yes	Yes	Yes	No	No	Yes	Yes	0.75	High
Strzelczyk, 2018 [37]	Yes	Yes	Yes	Yes	No	No	Yes	Yes	0.75	High
Schubert-Bast, 2018 [46]	Yes	Yes	Yes	Yes	No	No	Yes	Yes	0.75	High
Witt, 2018 [54]	Yes	Yes	Yes	Yes	No	No	Yes	Yes	0.75	High
Zahnert, 2018 [63]	Yes	Yes	Yes	Yes	No	No	Yes	Yes	0.75	High
Kalss, 2018 [70]	Yes	Yes	Yes	Yes	No	No	Yes	No	0.75	High
Willems, 2018 [74]	Yes	Yes	Yes	Yes	No	No	Yes	Yes	0.75	High
Menzler, 2019 [35]	Yes	Yes	Yes	Yes	No	No	Yes	Yes	0.75	High
Villanueva, 2019 [42]	Yes	Yes	Yes	Yes	No	No	Yes	Yes	0.75	High
Nissenkorn, 2019 [43]	Yes	Yes	Yes	Yes	No	No	Yes	Yes	0.75	High
Santamarina, 2019 [55]	Yes	Yes	Yes	Yes	No	No	Yes	Yes	0.75	High
Theochari, 2019 [64]	Yes	Yes	Yes	Yes	No	No	Yes	No	0.75	High
McGuire, 2020 [39]	Yes	Yes	Yes	Yes	No	No	Yes	Yes	0.75	High
Lafortune, 2020 [40]	Yes	Yes	Yes	Yes	No	No	Yes	Yes	0.75	High
Rene, 2020 [45]	Yes	Yes	Yes	Yes	No	No	Yes	Yes	0.75	High
Adewusi, 2020 [52]	Yes	Yes	Yes	Yes	No	No	Yes	Yes	0.75	High
Lerche, 2020 [61]	Yes	Yes	Yes	Yes	No	No	Yes	Yes	0.75	High
Steinhoff, 2020 [68]	Yes	Yes	Yes	Yes	No	No	Yes	Yes	0.75	High
Liguori, 2020 [69]	Yes	Yes	Yes	Yes	No	No	Yes	Yes	0.75	High
Fonseca, 2020 [73]	Yes	Yes	Yes	Yes	No	No	Yes	Yes	0.75	High
Ferratjans, 2021 [34]	Yes	Yes	Yes	Yes	No	No	Yes	Yes	0.75	High
Russo, 2021 [41]	Yes	Yes	Yes	Yes	No	No	Yes	Yes	0.75	High
Orlandi, 2021 [48]	Yes	Yes	Yes	Yes	Yes	No	Yes	Yes	0.87	High
Lattanzi, 2021 [50]	Yes	Yes	Yes	Yes	No	No	Yes	Yes	0.75	High
Stephen, 2021 [12]	Yes	Yes	Yes	Yes	No	No	Yes	Yes	0.75	High
Abraira, 2021 [60]	Yes	Yes	Yes	Yes	No	No	Yes	Yes	0.75	High
Savastano, 2021 [62]	Yes	Yes	Yes	Yes	No	No	Yes	Yes	0.75	High
Stefanatou, 2021 [66]	No	Yes	Yes	Yes	No	No	Yes	Yes	0.75	High
Depondt, 2021 [67]	Yes	Yes	Yes	Yes	No	No	Yes	Yes	0.75	High
Lattanzi, 2021 [71]	Yes	Yes	Yes	Yes	No	No	Yes	Yes	0.75	High
Strzelczyk, 2021 [72]	Yes	Yes	Yes	Yes	No	No	Yes	Yes	0.75	High
Lattanzi, 2022 [44]	Yes	Yes	Yes	Yes	No	No	Yes	Yes	0.75	High
Lattanzi, 2022 [47]	Yes	Yes	Yes	Yes	Yes	No	Yes	Yes	0.87	High
Russo, 2022 [51]	Yes	Yes	Yes	Yes	No	No	Yes	Yes	0.75	High
Green, 2022 [53]	Yes	Yes	Yes	Yes	No	No	Yes	Yes	0.75	High
Svendsen, 2022 [56]	Yes	Yes	Yes	Yes	No	No	Yes	Yes	0.75	High
Lattanzi, 2022 [58]	Yes	Yes	Yes	Yes	No	No	Yes	Yes	0.75	High
Lattanzi, 2022 [59]	Yes	Yes	Yes	Yes	No	No	Yes	Yes	0.75	High
Snoeren, 2022 [65]	Yes	Yes	Yes	Yes	No	No	Yes	Yes	0.75	High
Orlandi, 2023 [49]	Yes	Yes	Yes	Yes	No	No	Yes	Yes	0.75	High
Naddell, 2023 [57]	Yes	Yes	Yes	Yes	Yes	No	Yes	Yes	0.87	High

**Table 7:** Quality assessment using the JBI tool for quasi-experimental studies. JBI: Joanna Briggs Institute.

Author	Is it clear in the study what is the "cause" and what is the "effect" (i.e. there is no confusion about which variable comes first)?	Were the participants included in any comparisons similar?	Were the participants included in any comparisons receiving similar treatment/care, other than the exposure or intervention of interest?	Was there a control group?	Were there multiple measurements of the outcome both pre and post the intervention/exposure?	Was follow-up complete and if not, were differences between groups in terms of their follow-up adequately described and analyzed?	Were the outcomes of participants included in any comparisons measured in the same way?	Were outcomes measured in a reliable way?	Was appropriate statistical analysis used?	Total score/9	Quality interpretation
Yates, 2015 [80]	Yes	Yes	Yes	No	Yes	Yes	Yes	Yes	No	0.77	High
Strzelczyk, 2017 [77]	Yes	Yes	Yes	No	Yes	Yes	Yes	Yes	No	0.77	High
Liu, 2019 [78]	Yes	Yes	Yes	No	Yes	Yes	Yes	Yes	No	0.77	High
O’Brien, 2020 [81]	Yes	Yes	Yes	No	Yes	Yes	Yes	Yes	No	0.77	High
Arnold, 2020 [82]	Yes	Yes	Yes	No	Yes	Yes	Yes	Yes	No	0.77	High
Toledo, 2021 [79]	Yes	Yes	Yes	No	Yes	Yes	Yes	Yes	No	0.77	High
Ben-Menachem, 2021 [11]	Yes	Yes	Yes	No	Yes	Yes	Yes	Yes	No	0.77	High
Farkas, 2022 [76]	Yes	Yes	Yes	Yes	Yes	Yes	Yes	Yes	No	0.88	High
Chavarría, 2023 [83]	Yes	Yes	Yes	No	Yes	Yes	Yes	Yes	Yes	0.88	High
